# Neuron labeling with rhodamine-conjugated Gd-based MRI contrast agents delivered to the brain via focused ultrasound

**DOI:** 10.7150/thno.42665

**Published:** 2020-02-03

**Authors:** Sophie V. Morse, Tamara Boltersdorf, Bethany I. Harriss, Tiffany G. Chan, Nicoleta Baxan, Hee Seok Jung, Antonios N. Pouliopoulos, James J. Choi, Nicholas J. Long

**Affiliations:** 1 Department of Bioengineering, Imperial College London, South Kensington, London, SW7 2BP, UK.; 2 Department of Chemistry, Imperial College London, Molecular Sciences Research Hub, White City, London, W12 0BZ, UK.; 3 Biological Imaging Centre, Faculty of Medicine, Hammersmith Hospital, Du Cane Road, London, W12 0NN, UK.

**Keywords:** neurons, rhodamine, MRI contrast agents, focused ultrasound, blood-brain barrier

## Abstract

Gadolinium-based magnetic resonance imaging contrast agents can provide information regarding neuronal function, provided that these agents can cross the neuronal cell membrane. Such contrast agents are normally restricted to extracellular domains, however, by attaching cationic fluorescent dyes, they can be made cell-permeable and allow for both optical and magnetic resonance detection. To reach neurons, these agents also need to cross the blood-brain barrier. Focused ultrasound combined with microbubbles has been shown to enhance the permeability of this barrier, allowing molecules into the brain non-invasively, locally and transiently. The goal of this study was to investigate whether combining fluorescent rhodamine with a gadolinium complex would form a dual-modal contrast agent that could label neurons *in vivo* when delivered to the mouse brain with focused ultrasound and microbubbles.

**Methods**: Gadolinium complexes were combined with a fluorescent, cationic rhodamine unit to form probes with fluorescence and relaxivity properties suitable for *in vivo* applications. The left hemisphere of female C57bl/6 mice (8-10 weeks old; 19.07 ± 1.56 g; n = 16) was treated with ultrasound (centre frequency: 1 MHz, peak-negative pressure: 0.35 MPa, pulse length: 10 ms, repetition frequency: 0.5 Hz) while intravenously injecting SonoVue microbubbles and either the 1 kDa Gd(rhodamine-pip-DO3A) complex or a conventionally-used lysine-fixable Texas Red® 3 kDa dextran. The opposite right hemisphere was used as a non-treated control region. Brains were then extracted and either sectioned and imaged via fluorescence or confocal microscopy or imaged using a 9.4 T magnetic resonance imaging scanner. Brain slices were stained for neurons (NeuN), microglia (Iba1) and astrocytes (GFAP) to investigate the cellular localization of the probes.

**Results**: Rhodamine fluorescence was detected in the left hemisphere of all ultrasound treated mice, while none was detected in the right control hemisphere. Cellular uptake of Gd(rhodamine-pip-DO3A) was observed in all the treated regions with a uniform distribution (coefficient of variation = 0.4 ± 0.05). Uptake was confirmed within neurons, whereas the probe did not co-localize with microglia and astrocytes. Compared to the dextran molecule, Gd(rhodamine-pip-DO3A) distributed more homogeneously and was less concentrated around blood vessels. Furthermore, the dextran molecule was found to accumulate unselectively in microglia as well as neurons, whereas our probe was only taken up by neurons. Gd(rhodamine-pip-DO3A) was detected via magnetic resonance imaging *ex vivo* in similar regions to where fluorescence was detected.

**Conclusion**: We have introduced a method to image neurons with a dual-modal imaging agent delivered non-invasively and locally to the brain using focused ultrasound and microbubbles. When delivered to the mouse brain, the agent distributed homogeneously and was only uptaken by neurons; in contrast, conventionally used dextran distributed heterogeneously and was uptaken by microglia as well as neurons. This result indicates that our probe labels neurons without microglial involvement and in addition the probe was found to be detectable via both *ex vivo* MRI and fluorescence. Labeling neurons with such dual-modal agents could facilitate the study of neuronal morphology and physiology using the advantages of both imaging modalities.

## Introduction

Magnetic resonance imaging (MRI) can be used to acquire *in vivo* images of entire organisms noninvasively. In MRI images, tissues are generally distinguished based on differences in the concentration of water molecules and on the time taken for the protons in these water molecules to relax in a magnetic field. The contrast of magnetic resonance (MR) images can be enhanced by using intravenously-injected, paramagnetic contrast agents which are able to interact with nuclei adjacent or close to the paramagnetic centre 1. Relaxation enhancement of nuclei proximal to the paramagnetic centre depends on the electronic relaxation time of the metal 2. Gadolinium (Gd^3+^) complexes are the most commonly used for this purpose due to their uniquely long relaxation times (~10^-9^ s, several orders of magnitude slower than other lanthanide ions), their high magnetic moment (7.94 Bohr magnetons) and a free coordination site to directly bind to solvent water molecules. The interaction of gadolinium complexes with surrounding water molecules leads to a shortening of the longitudinal relaxation time (T_1_) of the water protons, resulting in a brightening of the image 1,3. Gd-based contrast agents can be used to investigate biological processes inside the body, such as developmental events, enzymatic activity and gene expression 4-7. However, most Gd-based contrast agents are restricted to extracellular domains as the cell membrane prevents the internalization of these highly hydrophilic agents 1. To tackle this issue, MRI contrast agents have been made membrane-permeable through the attachment of molecules such as cell-penetrating peptides (CPPs) 8-13. CPP-conjugated complexes, however, leak easily from cells 14 and lead to quenched MRI signals due to endosomal or lysosomal entrapment 15. Another approach is to attach fluorescent dyes to Gd^3+^ complexes (such as rhodamine, fluorescein isothiocyanate (FITC), cyanine 7 (Cy7) and boron dipyrromethene (BODIPY)) 5,16,17. These dyes cross the cell membrane easily by themselves but can also make other complexes more cell permeable by being conjugated to them. Fluorescent dyes are advantageous for this purpose as they are cheaper than CPPs, easier to conjugate and provide an additional means of imaging the probes using fluorescence microscopy as well as MRI.

In the field of neuroscience there is a growing need for methods to understand the neural system *in vivo*. One area of interest is the labeling of neurons to facilitate the study of neuronal morphology and their function. Unique information regarding neuronal physiology, such as metabolic activity and protein expression, can be provided by MRI contrast agents that are capable of crossing the cell membrane 18,19. Rhodamine B is a commonly used fluorophore that is biocompatible, possesses a high quantum yield and has been used to label neurons *in vivo* 20-22. This dye has been reported to be non-toxic to neurons, resistant to histological fixation procedures and does not leak from labeled cells 20. Rhodamine constructs have been previously conjugated with Gd-based MRI contrast agents to permeate cells 5,16. Amongst these, Gd^3+^-rhodamine complexes based on a macrocyclic, DOTA (tetraazacyclododecane-1,4,7,10-tetraacetic acid) scaffold (Gd(Rhoda-DOTA)) have been reported to enter HeLa cells *in vitro* 16, Xenopus embryos *in vivo* 5, and have also been used as dual-modal probes (Gd(rhodamine-DO3A)) to image tumours in mice 23. Rhodamine B and similar motifs can interconvert between a spirocyclic non-fluorescent form and a fully conjugated ring-opened form upon activation of the carbonyl group, a property that has been extensively investigated in the context of pH and heavy metal ion sensing 24,25. Here, we have modified the structure of rhodamine B to give an “always on” fluorescent probe by adding a piperazine unit to the amide to lock it in its ring-opened form, while creating a positive charge on the dye to facilitate cell permeability. Next, the rhodamine derivative is combined with a macrocyclic gadolinium complex to form a compound that can enable both the accumulation of a Gd-based contrast agent in neurons and simultaneous fluorescence imaging. To the best of our knowledge, a similar probe has not yet been used to image neurons and such a compound could allow for simultaneous imaging of neurons at different resolutions and depths in preclinical models, combining the advantages of fluorescence imaging with those of MRI.

In order to effectively image neurons, the probe must also cross the blood-brain barrier (BBB). The BBB prevents most molecules above 400 Da from entering the brain to maintain homeostasis and protect the brain from toxic substances 26,27. Therefore, under normal conditions where the BBB is intact, our complex cannot enter the brain 28. Direct injection through the skull can be used to deliver these complexes, however, this is an invasive procedure which can lead to complications such as haemorrhage and infection 29,30. A non-invasive alternative to deliver these compounds across the BBB is to use focused ultrasound and microbubbles 31. With this method, ultrasound is focused onto a specific region of interest where the compound is to be delivered. Clinically approved microbubbles are then injected intravenously, and when they reach the region where the ultrasound is focused, the microbubbles expand and contract. This mechanical stimulation of the vessels increases permeability and allows compounds to cross the BBB. A wide range of compounds have been delivered using this technique, including Gd-based MRI contrast agents 31-33, fluorescent dextrans 34-36, nanoparticles 37,38, drugs 39,40, peptides 41 and antibodies 42,43. This ultrasound technology has previously been used to deliver fluorescent dextrans into neurons 35,44, however, it has not been used for neuronal delivery of optical-MRI probes. To date, focused ultrasound combined with microbubbles is the only non-invasive, localized and transient way of getting these compounds into deep regions of the brain.

In this study, we report a way of imaging neurons with a dual-modal imaging agent delivered to the brain with focused ultrasound and microbubbles. Gd(rhodamine-pip-DO3A) was prepared and its optical and relaxivity properties were characterized. The compound was delivered noninvasively to the left hemisphere of mice using focused ultrasound and microbubbles. To examine the distribution of the compound within the parenchyma, fluorescence images were acquired. Its cellular uptake was compared to that of an optical probe (Texas-Red dextran) normally used to assess BBB permeability enhancement following focused ultrasound-mediated delivery. *Ex vivo* MRI images of the brains were then taken to evaluate the presence of the compound. The delivery of such dual-modal agents into neurons could facilitate the study of neuronal morphology and physiology using both MRI and fluorescence microscopy across different spatiotemporal scales.

## Methods

### General synthetic and spectroscopic methods

Reagents were purchased and used without further purification from Sigma Aldrich, Fisher Scientific or Goss Scientific. To characterize the different steps of the probe's synthesis, NMR, MALDI and fluorescence spectra were acquired. Proton and carbon nuclear magnetic resonance (^1^H-NMR; ^13^C-NMR) spectra were recorded at room temperature on a Bruker AMX-400 spectrometer. Chemical shifts in the NMR spectra are reported in parts per million (ppm) with coupling constants quoted in hertz (Hz) to the nearest decimal point. Multiplicities are abbreviated as follows: s = singlet, d = doublet, t = triplet, q = quartet, m = multiplet, br = broad, sbr = broad singlet. Electrospray ionization (ES+) mass spectra were collected on a Waters LCT Premier spectrometer. MALDI spectra were collected on a low resolution Micromass MALDI-ToF machine. Fluorescence spectra were recorded on a Varian Cary Eclipse fluorescence spectrophotometer using quartz cuvettes. T_1_ measurements were performed on a Bruker DRX-400 spectrometer. The complexes were dissolved in H_2_O at five different concentrations and placed in 1.7 mm diameter capillary tubes, sealed with Parafilm. These were placed in 5 mm NMR tubes and filled with D_2_O and 1/ T_1_ measurements were performed. The concentration of Gd^3+^ in these samples was confirmed by measuring the chemical shift difference between HOD and H_2_O signals induced by the paramagnetic Gd^3+^ at 25°C on a Bruker AV 500 45. Synthetic and spectral details are given in the supplementary information.

### Animals

Sixteen female wild-type C57bl/6 mice (8-10 weeks old, 19.07 ± 1.56 g; Envigo, Huntingdon, UK) were used in this study. An acclimatization period of seven days was allowed prior to the initiation of any procedure and all experimental protocols were approved by the institutional animal facility committee and the UK Home Office regulatory establishments.

### Ultrasound setup and experimental conditions

**Figure 1:**
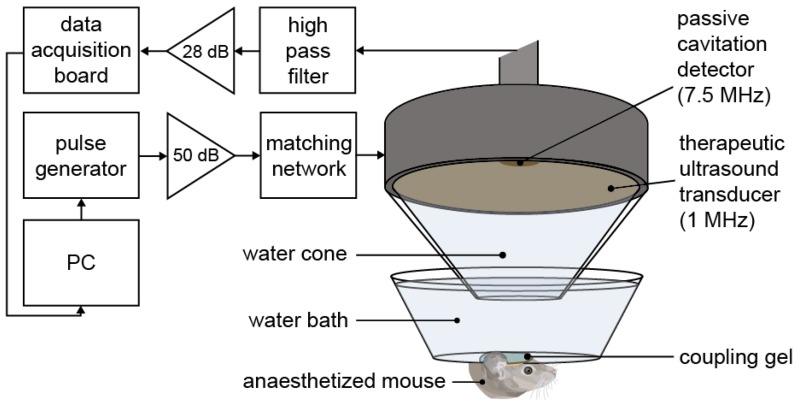
** Ultrasound experimental setup.** The mouse brain was exposed to 1-MHz ultrasound using a ms-long pulse sequence, composed of 10,000-cycle pulses emitted at a slow rate of 0.5 Hz at a peak negative pressure (P_neg_) of 0.35 MPa. Ultrasound was emitted onto the left hemisphere through the intact scalp and skull, while the right hemisphere was used as a control, with no ultrasound focused onto it. A 7.5-MHz passive cavitation detector (PCD) was used to detect the microbubble signals to verify their presence. (PC = personal computer, dB values refer to the amount of amplification).

Mice were anaesthetized with 1.5-2.0% vaporized isoflurane (Zoetis UK Limited, London, UK) mixed with oxygen (0.8 L/min) using an anesthesia vaporizer (Harvard Apparatus, Cambridge, UK). The fur was first removed from the mouse's head with an electric trimmer and depilatory cream, and then the head was placed within a stereotaxic frame (45° ear bars; World Precision Instruments, Hertfordshire, UK). After applying ultrasound gel to the mouse head, a container covered by a transparent parafilm membrane filled with degassed water was placed on the gel, making the sutures of the skull clear for targeting purposes. The ultrasound transducer, mounted with a cone filled with distilled water covered by an acoustically transparent parafilm membrane, was lowered into the container (Figure 1). For targeting purposes, a 1 mm thick metal cross was aligned to the lambdoid and sagittal sutures of the skull at the bottom of the water container 46. The transducer was positioned 3 mm laterally from the sagittal suture, 0.5 mm anterior to the lambdoid suture and 3 mm inferior to the skull to target the left hemisphere of the brain with the ultrasound focus above the left hippocampus 44. This brain region was chosen due to its potential as a therapeutic target and due to the low acoustic attenuation of the parietal bone. The opposite right hemisphere was used as a control (no ultrasound treatment) in all experiments. For this targeting procedure, the ultrasound transducer was used in pulse-echo mode, with the transducer connected to a pulser-receiver (DPR300; Insidix, Seyssins, France) and moved by a 3D computer-controlled positioning system (Velmex Inc., Bloomfield, NY, USA).

After targeting, therapeutic ultrasound pulses were emitted from the single-element spherical-segment focused ultrasound transducer (centre frequency: 1 MHz, focal depth: 60.5 mm, diameter: 90 mm; Sonic Concepts, Bothell, WA, USA). The pulses were emitted from a function generator (33500B Series; Agilent Technologies, Santa Clara, CA, USA) and passed through a 50-dB power amplifier (Precision Acoustics Ltd, Dorchester, UK) before reaching the transducer. Ultrasound pulses were emitted for all experiments: peak-negative pressure = 0.35 MPa_pk-neg_, pulse length = 10 ms, pulse repetition frequency = 0.5 Hz, number of pulses = 125. Prior to the *in vivo* experiments, the pressure amplitude reported in this study was measured with a needle hydrophone (needle diameter: 0.2 mm, Precision Acoustics Ltd., Dorchester, Dorset, UK) in a degassed water tank. The peak-positive and peak-negative values were calculated and attenuated by 11.2 ± 3.2 % to correct for the skull attenuation, which was measured experimentally through the parietal bone of the mouse's skull (n = 4). The axial, lateral and elevational full width at half maximum intensities of the ultrasound beam were 20 mm, 2 mm and 1 mm respectively.

During the ultrasound treatment, a passive cavitation detector (PCD, centre frequency: 7.5 MHz, focal length: 76.2 mm; Olympus Industrial, Essex, UK) was used to detect the microbubble signals to verify their presence and behavior in the targeted region. This detector was positioned through the central opening in the therapeutic transducer with the foci aligned and overlapping. These acoustic emissions were filtered by a 3-30 MHz band-pass filter (Mini circuits, Brooklyn, NY, USA) amplified by a 28 dB pre-amplifier (Stanford Research Systems, Sunnyvale, CA, USA) to then be recorded by a 8-bit oscilloscope sampling at 250 MHz (Picoscope 3205A; Pico Technology, Cambridgeshire, UK). The acoustic emissions were processed in Matlab (Mathworks, Natick, MA, USA) to evaluate the energy levels compared to those of the control pulses.

### Microbubbles and probe delivery

Ten seconds into the ultrasound sonication, allowing five control pulses to be emitted, SonoVue® microbubbles (Bracco, Milan, Italy) were administered intravenously through a 30 G catheter over a 30 s time period (volume: 100 µl, concentration: 5 µl/g). The microbubbles were activated following manufacturers' instructions and were used within 6 h from activation. One minute from the start of the sonication, Gd(rhodamine-pip-DO3A) (molecular weight: 1 kDa, concentration: 5.6 mg/ml; n = 10) or lysine-fixable Texas Red® 3 kDa dextran (concentration: 5 mg/ml; Life Technologies, Paisley, UK; n = 6) was also injected. The probes were diluted in 100 µl phosphate-buffered saline (PBS) and were not expected to cross an intact BBB due to their size being over the 400 Da threshold 47. Of the ten mice where Gd(rhodamine-pip-DO3A) was delivered, three brains were imaged with MRI as well as fluorescence microscopy.

### Histological staining

Following ultrasound treatment, the mice were euthanized with 20 mL PBS and 20 mL 10% formalin (Sigma Aldrich, St Louis, MO, USA). The brain was extracted from the skull and fixed in formalin overnight, followed by 15% sucrose for 6 hours and 30% sucrose overnight to protect the tissue before frozen sectioning. The brains were embedded in optimal cutting temperature (OCT; Agar Scientific, Stansted, UK) compound and sectioned into sixty 30 µm slices to cover the entire hippocampus using a cryostat (CryoStar NX70; Thermo Fisher, Waltham, MA, USA).

Immunohistochemistry (IHC) of 12 frozen sections from each brain was used to determine which cells (neurons, microglia or astrocytes) were taking up the probes. For neuronal staining: primary recombinant anti-NeuN antibody (1:500 overnight; Ab177487; Abcam, Cambridge, England) and secondary goat anti-rabbit IgG H&L Alexa Fluor® 488 antibody (1:500 for 2 h; Ab150077; Abcam); for microglia staining: primary anti-Iba1 antibody (1:500 overnight; Ab5076; Abcam) and secondary donkey anti-goat IgG H&L Alexa Fluor® 488 antibody (1:500 for 2 h; Ab150129; Abcam); for astrocyte staining: primary GFAP monoclonal antibody (1:50 overnight; 13-0300; ThermoFisher) and secondary mouse anti-rat IgG2a FITC antibody (1:500 for 2 h; 11-4817-82; ThermoFisher).

### Microscopy and analysis

Images of the brain slices with the delivered probes and the antibody staining were acquired using fluorescence microscopy (10x; Zeiss Axio Observer; Oberkochen, Germany) and confocal microscopy (20x; Zeiss LSM-510 inverted; Oberkochen, Germany). Gd(rhodamine-pip-DO3A) and Texas Red 3 kDa dextran were excited at 562/40 nm and emissions were filtered at 624/40 nm. The Alexa Fluor 488 and FITC stained slices were excited at 470/40 nm and emissions were filtered at 525/50 nm.

The detected dose was measured with the normalized optical density (NOD) 35. All pixels with intensities higher than the mean of the control region plus twice its standard deviation were summed for both the control and targeted regions of interest. The sum of the targeted region was subtracted by that of the control region to obtain the NOD. The distribution of the probes was quantified with the coefficient of variation (COV), defined as the standard deviation over the average fluorescence intensity in the targeted region. This was calculated for six slices for each treated brain by selecting regions of interest around the targeted left hippocampus using Matlab® (2016a, The Mathworks, Natick, MA, USA).

### Fluorescence Titration with Bovine Serum Albumin

The following binding assay was performed to assess whether Gd(rhodamine-pip-DO3A) might interact with endogenous albumin in the blood. Such binding can slow down the motion of the agent, thereby enhancing r_1_ relaxivity 48. A solution of 10 μM bovine serum albumin (BSA) was titrated with increasing concentrations of Gd(rhodamine-pip-DO3A) (1 μM to 10 μM; 0.1 to 1 eq.). After each addition, the fluorescence emission between 310-450 nm was recorded using a Cary Eclipse Fluorescence Spectrophotometer with an excitation wavelength of 295 nm. Emission values at 345 nm (λ_max_) were normalized to the emission recorded for BSA without any complex added. All samples were measured in triplicate. Quenching constants (K_sv_) were calculated by plotting Stern-Volmer plots (I_0_/I = K_sv_[X] + 1, where I = intensity and [X] = complex concentration).

### Ex vivo MRI

**Figure 2:**
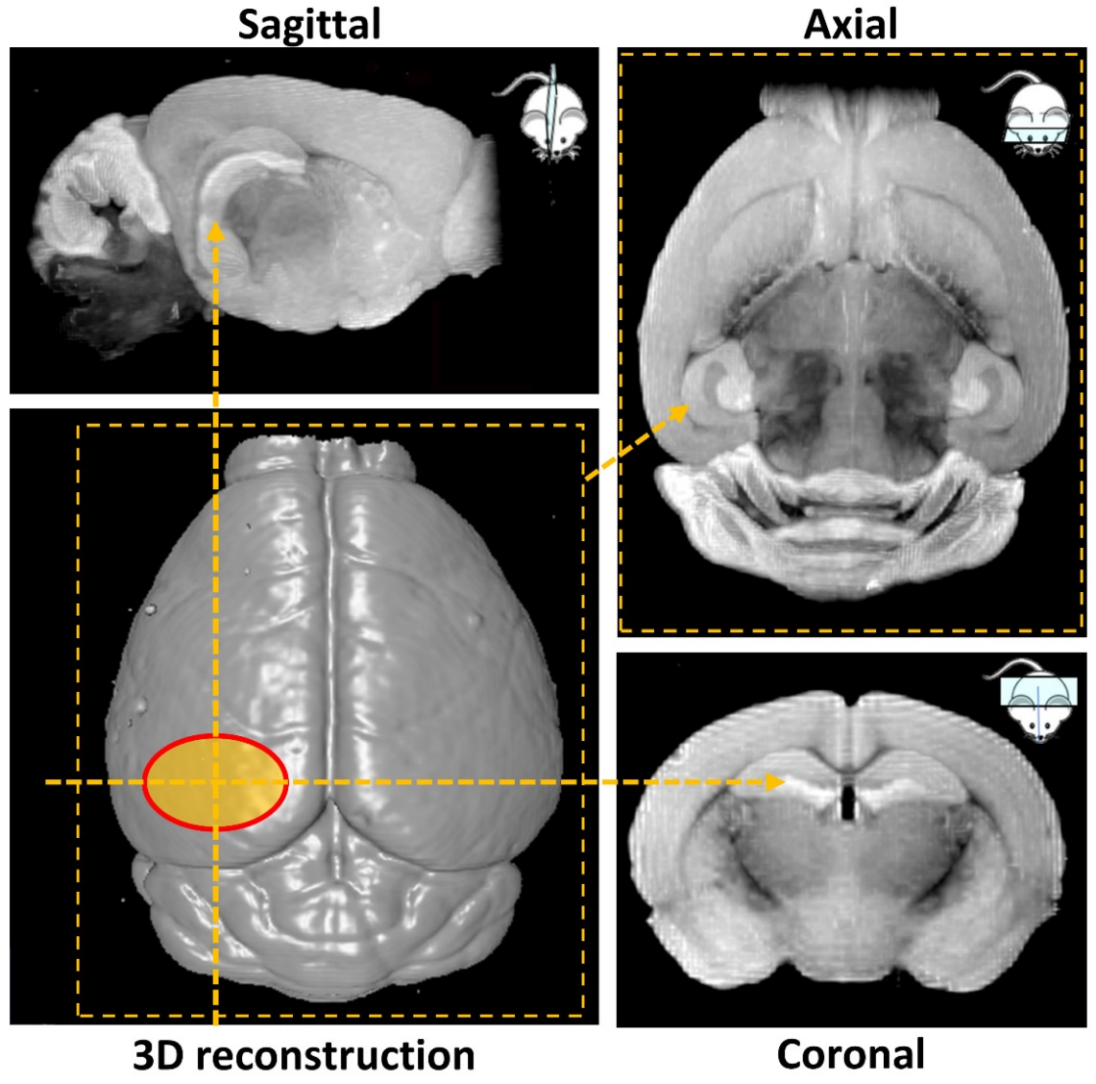
MRI-based 3D volume reconstruction of the mouse brain highlighting the region where ultrasound targets the left hemisphere (red oval). Maximum intensity projections (MIP) over a volume of 1 mm thickness highlight the targeted hemisphere in sagittal, axial and coronal orientations (yellow arrows).

*Ex vivo* MRI scans were performed at Imperial College Biological Imaging Centre using a pre-clinical 9.4 T scanner (94/20 USR Bruker BioSpec; Bruker Biospin, Ettlingen, Germany) equipped with a 40 mm inner diameter volume transmit/receive quadrature coil. Data was acquired with Paravision 6.0.1 (Bruker, BioSpin). T_1_ weighted images were obtained with a 3D gradient echo-based FLASH sequence with the field of view selected to cover the entire mouse brain. Additional acquisition parameters were: T_R_/T_E_ = 50/7.2 ms (T_R_ = repetition time; T_E_ = echo time), flip angle = 32°, spatial resolution = (100×100×100) µm^3^, 14 averages, total scan time 7 h. 3D volume reconstruction and smoothing was performed in Paravision 6.0.1 to illustrate the surface of the mouse brain and the targeted hippocampal area (Figure 2). Next, maximum intensity projection was carried out over a volume of 1 mm thickness to highlight the targeted left hemisphere and its contralateral side in sagittal, axial and coronal orientation (Figure 2). The signal intensity on the targeted side was compared to the control side by calculating the normalized signal intensity, using the same calculation as used for the NOD.

### Statistical analysis

A two-sided Student* t* test was performed to determine whether the COV values were significantly different between the brains where Gd(rhodamine-pip-DO3A) and dextran were delivered. A value of p < 0.01 was considered statistically significant.

## Results

### Synthesis of Gd(rhodamine-pip-DO3A)

**Figure 3:**
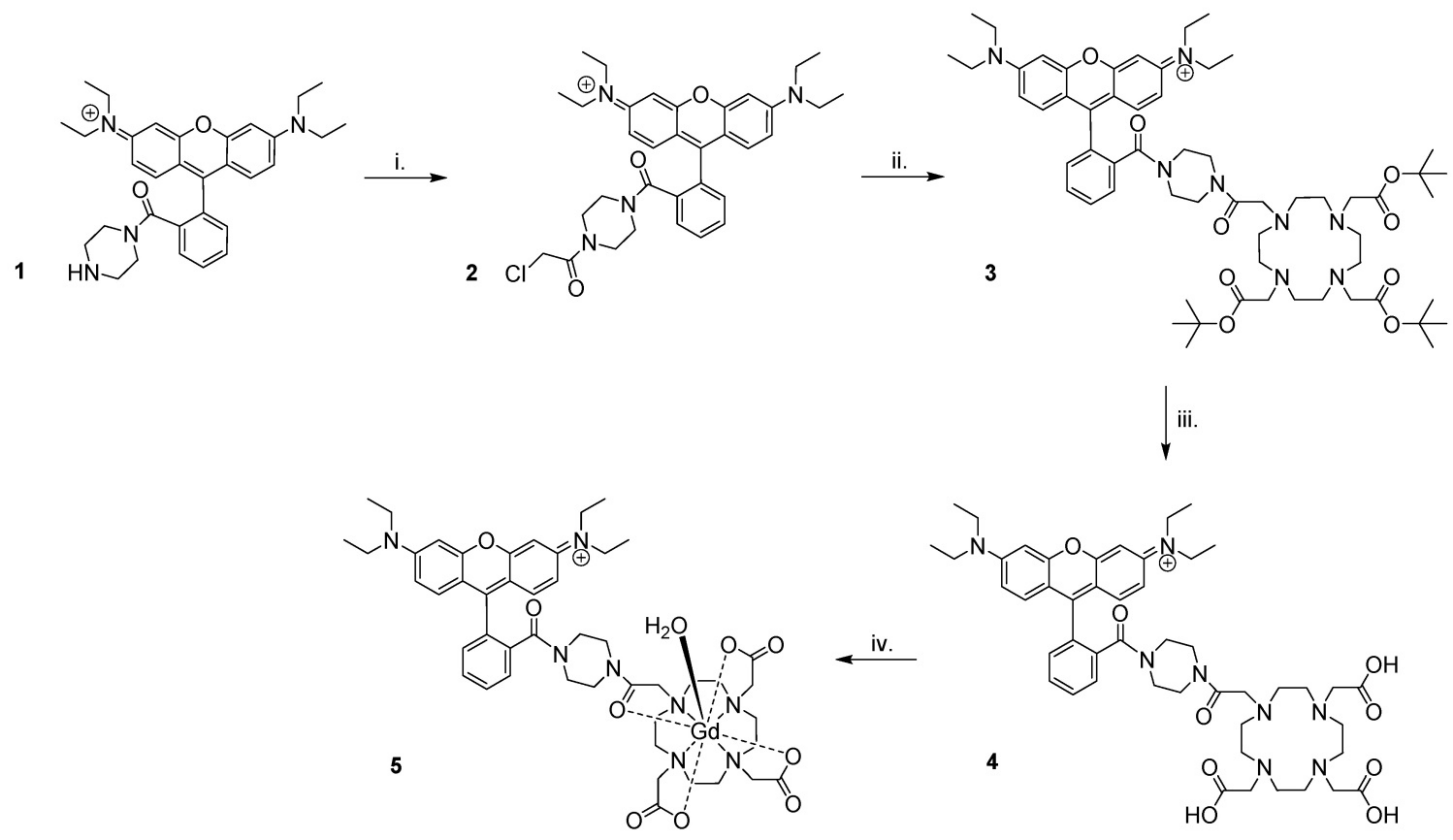
** Synthesis of Gd(rhodamine-pip-DO3A).** The following reaction conditions were used: i. Chloroacetyl chloride, NEt_3_, DCM (dichloromethane), 3 h, 0 °C, 62% yield; ii. 1,4,7,10-tetraazacyclododecane-1,4,7-tris(t-butyl acetate), K_2_CO_3_, CH_3_CN, 48 h, 82 °C, 66% yield; iii. TFA (trifluoroacetic acid), DCM, 16 h, room temperature (RT), 74% yield; iv. GdCl_3_.6H_2_O, pH 5.5, 24 h, RT, 65-87% yield.

Preparation of Gd(rhodamine-pip-DO3A) (**5**) was achieved as summarized in Figure 3. Briefly, rhodamine B was treated with 1-boc-piperazine to form a tertiary amide and thus lock the rhodamine unit into its ring-opened, fluorescent form. Hence, the compound was prevented from undergoing spirocyclisation 49, while forming a cationic species at the same time. The resulting compound was purified by column chromatography and deprotected 50 to produce compound **1**. Next, chloroacetyl chloride was added under basic conditions to obtain compound** 2**, before further reaction with *tert*-butyl protected DO3A to form compound **3**. Cleavage of the ester groups resulted in isolation of **4** and subsequent lanthanide complexation afforded compound **5** in good yields (65-87%) after purification using reverse phase column chromatography.

### Fluorescence and relaxometric measurements and determination of r_1_

**Figure 4:**
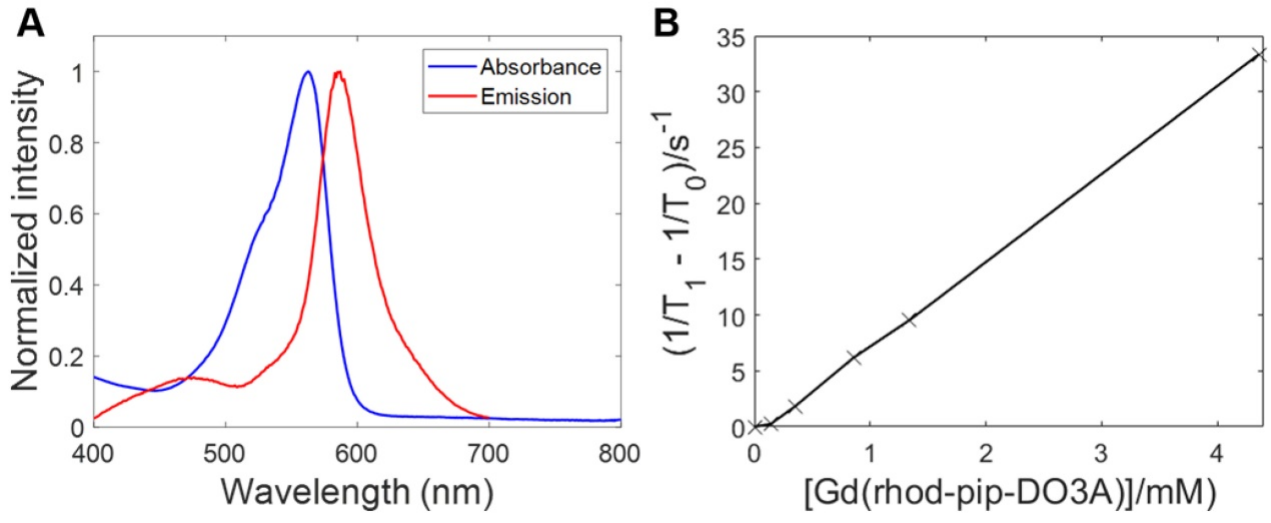
** Fluorescence excitation and emission spectra of Gd(rhodamine-pip-DO3A) and T_1_ relaxation measurements to determine relaxivity (r_1_). (A)** Absorbance and emission spectra of Gd(rhodamine-pip-DO3A) show typical rhodamine maxima (in PBS at pH 7.3, λ_exc_ = 360 nm). **(B)** The change in longitudinal relaxation with increasing concentration of Gd(rhodamine-pip-DO3A) was used to determine relaxivity (r_1_). T_1_ represents the relaxation time measured with the contrast agent present while T_0_ represents the relaxation time measured in water alone without the contrast agent.

To determine the optical properties of the probe, the normalized absorption and emission spectra of Gd(rhodamine-pip-DO3A) were obtained in PBS solution at pH 7.3 and are depicted in Figure 4A. Typical rhodamine-based maxima were observed within the absorption spectrum at 562 nm and at 587 nm (λ_exc_ = 360 nm) within the emission spectrum and indicated that the compound was suitable to use for *in vivo* optical applications.

As has been described above, Gd^3+^ chelates are known to affect T_1_ relaxation times of surrounding water molecules and as a result can be used to alter the contrast in MRI images, providing a clearer image of physiological changes 51. To quantify the degree to which a contrast agent alters the water proton relaxation rates (1/T_1_) of nearby solvent molecules, relaxivity values are used. This parameter is defined as the change in relaxation rate in the presence of the contrast agent divided by the concentration of the metal ion 1,51. To determine the longitudinal relaxivity value (r_1_) of Gd(rhodamine-pip-DO3A) and thus predict whether it would act as a useful MRI contrast agent in a physiological context, samples were prepared at five different molar concentrations. Each of their gadolinium concentrations was subsequently determined by ^1^H-NMR spectroscopy by making use of the chemical shift difference between HOD and H_2_O signals induced by the paramagnetic Gd^3+^ 45. Next, T_1_-value measurements at well-defined molar concentrations were performed, and the r_1_ value was determined as 7.8 mM^-1^s^-1^ at 400 MHz (25°C) (Figure 4B) 52.

This relaxivity value is higher than that expected for octadentate gadolinium chelates, therefore, we sought to investigate what this could be due to. In solution, lanthanide DOTA complexes are present as two diastereomers that can interconvert, known as the twisted square antiprism (TSAP) isomer and the square antiprism (SAP) isomer 53,54. Faster water exchange, and thus increased relaxivity values, is often associated with the minor TSAP DOTA isomer 54,55, whereas the major SAP isomer is less beneficial for relaxivity factors.

To approximate the SAP to TSAP ratio and associated water exchange properties in our system, ^1^H-NMR-spectroscopy is a convenient method to use, as the chemical shift of axial ring protons in the TSAP and SAP isomers are significantly separated 56. However, due to very large shift ranges and strong line broadening effects, most lanthanide complexes cannot be visualized easily with ^1^H-NMR spectroscopy 57. In Eu^3+^ compounds, these effects are minimized, making them the most convenient analogues for ^1^H-NMR spectroscopy 58. To investigate the ratio between the two isomers, we therefore prepared the europium analogue of our Gd probe (Eu(rhodamine-pip-DO3A), Figure S1) and were able to approximate 59 a SAP to TSAP ratio of 1:0.56 by relative integration.

### Ultrasound-mediated delivery and neuronal uptake of Gd(rhodamine-pip-DO3A)

**Figure 5:**
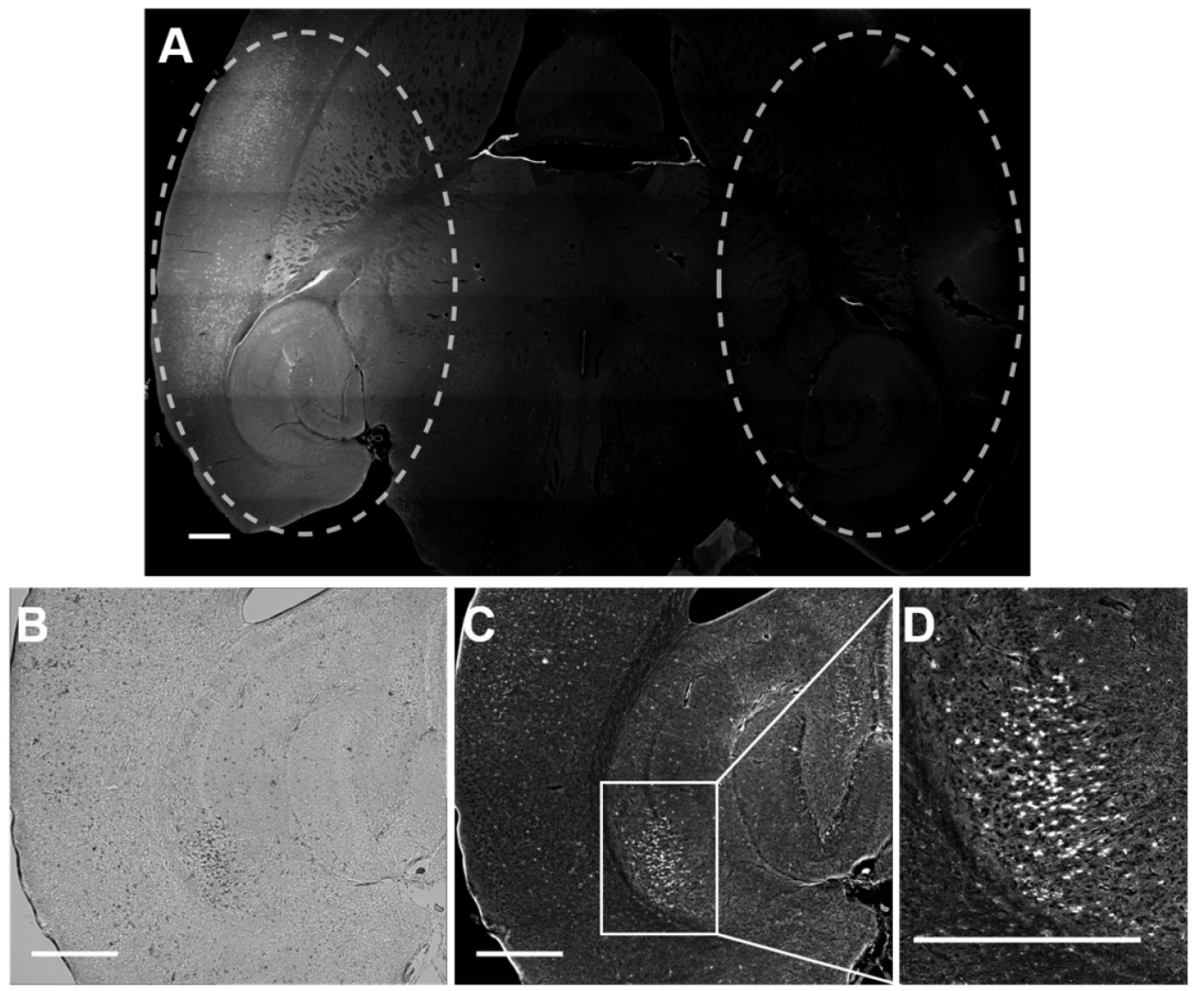
** Delivery and cellular uptake of Gd(rhodamine-pip-DO3A) in the left hemisphere of the mouse brain. (A)** The fluorescence image (10x) shows where Gd(rhodamine-pip-DO3A) was detected in the left hemisphere (left dashed circle) while no fluorescence was detected in the right hemisphere (right dashed circle) where no ultrasound was present (control region). The location of the probe is shown by all the regions where there is high intensity in the fluorescence image (white). **(B)** This bright field image shows the left hippocampus where the probe was delivered, where the dark spots highlight the location of cells within the selected region. **(C-D)** In most of these cells, fluorescence is detected indicating that Gd(rhodamine-pip-DO3A) is being uptaken. **(D)** This zoomed in region highlights an area with high cellular uptake. The white scale bar indicates 500 µm.

Rhodamine fluorescence was detected in the left hemisphere of all mice treated with focused ultrasound, while in the right hemisphere, where no ultrasound was present, no fluorescence was detected (Figure 5). This result confirmed that Gd(rhodamine-pip-DO3A) does not cross the BBB unless ultrasound is applied in combination with circulating microbubbles. Cellular uptake of Gd(rhodamine-pip-DO3A) was observed in all mouse brains (Figure 5A, C-D). The dark spots observed in the bright field images (Figure 5B) show where cells are present. Most of these dark spots correspond to regions where fluorescence signal was also observed, indicating that the probe is being uptaken by many of the cells in the region where the ultrasound is focused.

**Figure 6:**
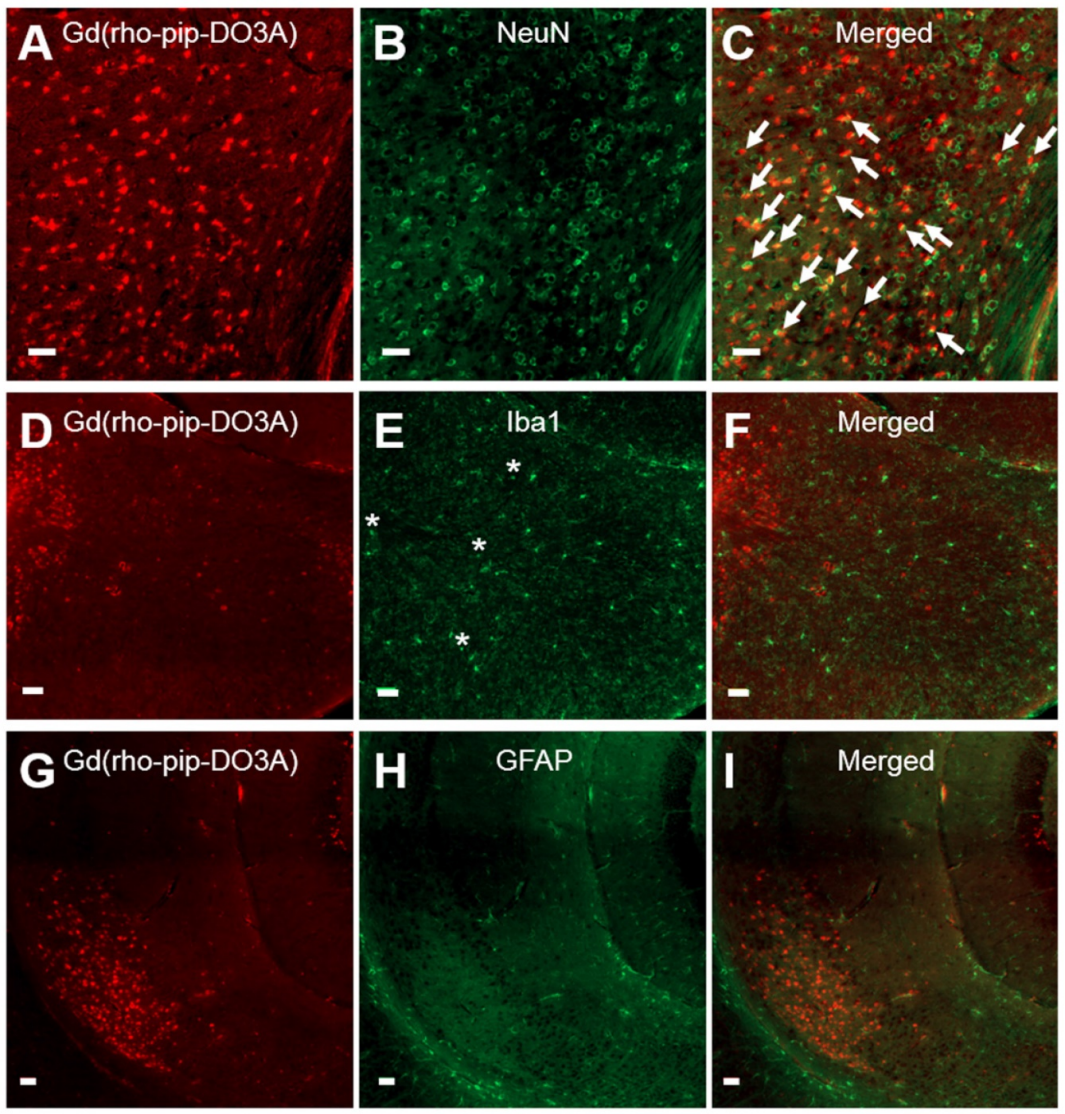
** Neuronal, microglial and astrocyte staining of brain slices with Gd(rhodamine-pip-DO3A) delivery**. Fluorescence images (10x) of Gd(rhodamine-pip-DO3A) distribution (A, D, G) and of immunohistological staining for neurons using NeuN (B), microglia using Iba1 (E) and astrocytes using GFAP (H) with respective merged channels (C, F, I). The white arrows indicate points of colocalisation between the probe and neuronal staining and the asterisks are positioned above more rounded microglia with shorter processes, indicating possible activation. No co-localization was observed between the probe and microglia or astrocytes. The white scale bars indicate 50 µm.

Based on cellular morphology, uptake of the probe was only observed in neuronal-like cells which was confirmed by immunohistological staining (Figure 6 A-C). No uptake, however, was detected in microglia (Figure 6 D-F) or astrocytes (Figure 6 G-I). The number of neurons overlapping with our Gd(rhodamine-pip-DO3A) probe was found to be significantly different from the number of microglia and astrocytes overlapping with the probe (*p < 0.01*; Figure S2). Although no co-localization was observed, some microglia in the targeted regions appeared more rounded with shorter processes, indicating that they may be in an active form. Lastly, only small signs of red blood cell extravasation and microvacuolations were observed in one of the three brains stained for H&E (haematoxylin & eosin; Figure S3).

### Distribution comparison of Gd(rhodamine-pip-DO3A) with Texas Red dextran

**Figure 7:**
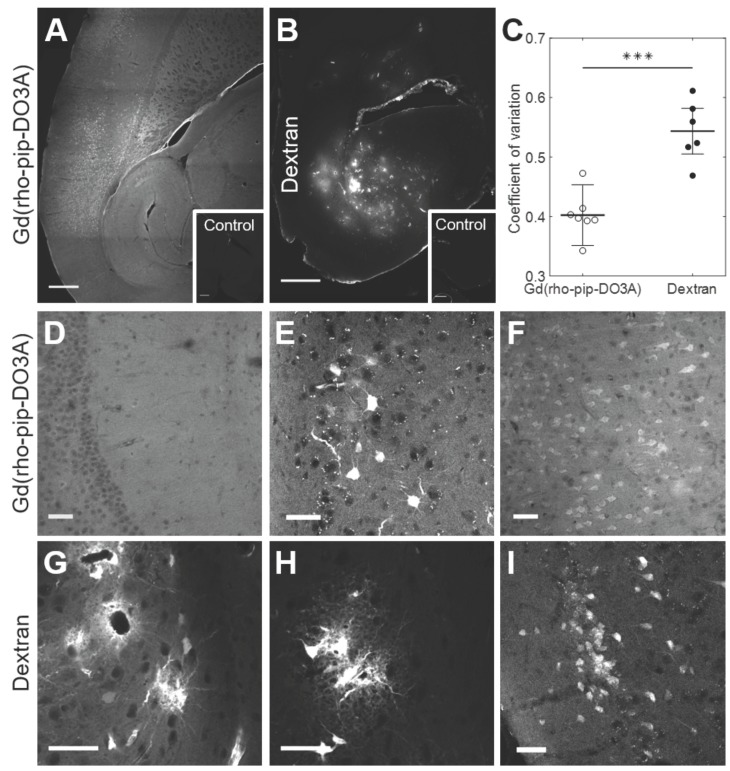
** Distribution of Gd(rhodamine-pip-DO3A) and dextran within the brain.** Fluorescence images (10x) show the distribution of (**A**) Gd(rhodamine-pip-DO3A) and (**B**) Texas Red-Dextran in the left hemisphere of the brain. Corresponding right hippocampi, which were control untreated regions, are displayed in smaller white boxes on the bottom right of each image. No fluorescence was detectable in these regions. The white scale bar indicates 500 µm. (**C**) A significant difference in the coefficient of variation (COV), a measure of distribution heterogeneity, was quantified (n = 6; P < 0.001). The distribution of Gd(rhodamine-pip-DO3A) was more homogeneous than that of dextran. **(D-I)** Confocal microscopy images (20x) of mouse brain regions exposed to ultrasound reveal (**D**) Gd(rhodamine-pip-DO3A) delivered homogeneously throughout the parenchyma with (**E-F**) cell uptake. (**G**) Dextran instead accumulated mainly around the blood vessels and was also taken up by (**H**) microglial-looking cells and (**I**) neuronal cell bodies. The white scale bars in these confocal images indicate 50 µm.

The distribution and cellular uptake of our 1 kDa Gd(rhodamine-pip-DO3A) probe (**5**) within the brain was compared to that of a larger molecule, Texas Red 3 kDa dextran, often used to assess the permeability enhancement of the BBB following focused ultrasound and microbubble treatment 34-36. Gd(rhodamine-pip-DO3A) was found to distribute uniformly within the targeted area (Figure 7A, D), while a more heterogeneous pattern was observed when the dextran was delivered (Figure 7B, G). The distribution was quantified using the coefficient of variation (COV) defined as the standard deviation over the average fluorescence intensity in the targeted region. The COV showed that there is less variation in the distribution of the dual-modal probe (COV = 0.4 ± 0.05) compared to the dextran (COV = 1.23 ± 0.04), providing a better coverage of the tissue (Figure 7C). Gd(rhodamine-pip-DO3A) was also detected across a much larger region of the brain compared to the dextran (Figure 7A-B).

By taking a closer look with confocal microscopy, the synthesized probe not only had a more uniform distribution (Figure 7D), with no accumulation around the blood vessels, but was also uptaken by cells throughout the ultrasound focus (Figure 7E-F). Interestingly, the probe was observed both within the nucleus and cytoplasm of cells with some high intensity regions (approximately 1-3 µm; Figure S4). The dextran, on the other hand, accumulated around the blood vessels (Figure 7G) and was also uptaken by cells, however, these cells were not only neuronal-like cells (Figure 7I) but also glial-like cells (Figure 7G-H). Immunohistochemistry confirmed the uptake of dextran within neurons and more so within microglia but not in astrocytes (Figure S5), while Gd(rhodamine-pip-DO3A) was only found in neurons. With both probes, more rounded microglia with shorter processes were detected in the ultrasound-targeted regions.

### Detection of Gd(rhodamine-pip-DO3A) with MRI

**Figure 8:**
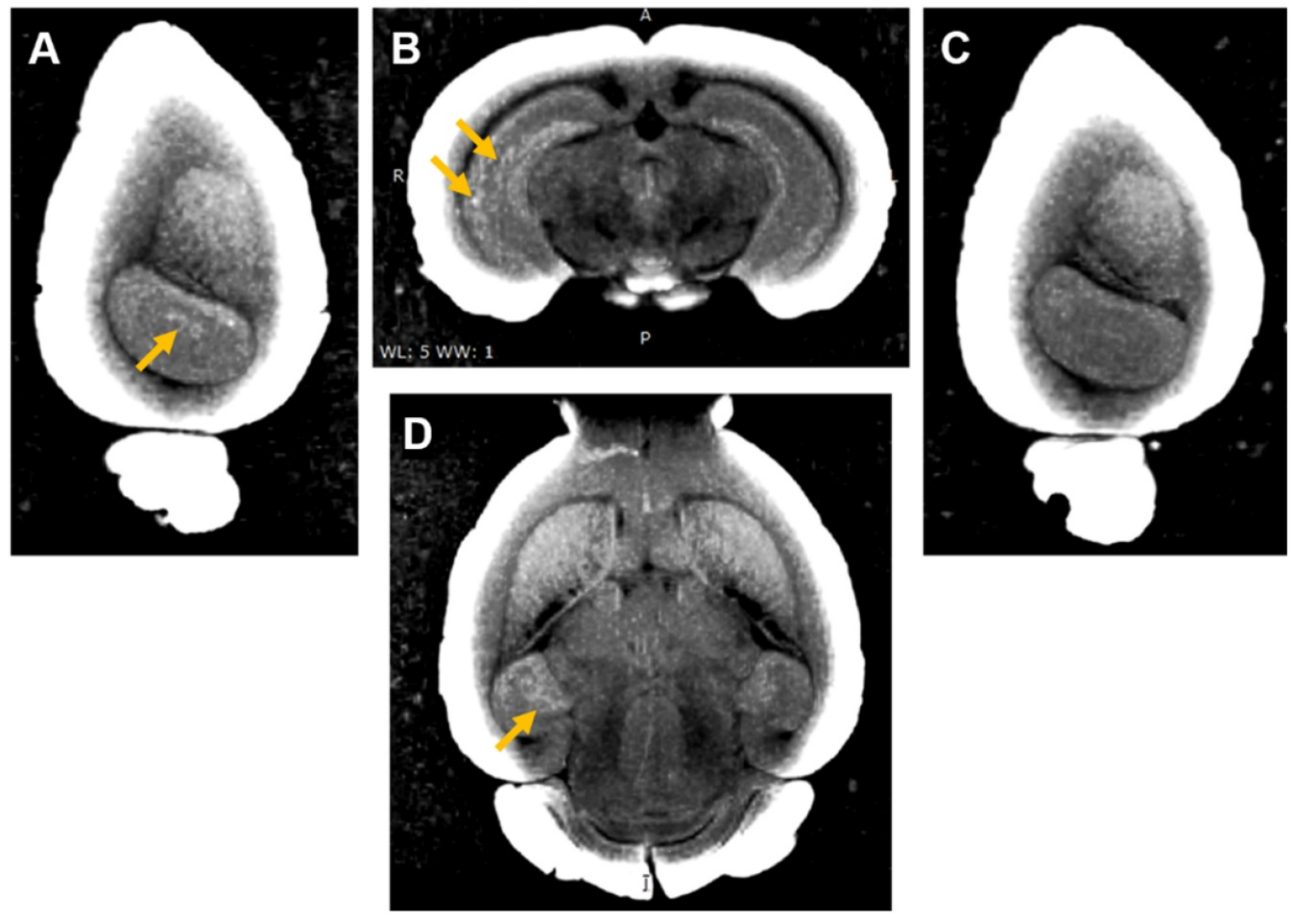
***Ex vivo* MRI images of brain with Gd(rhodamine-pip-DO3A) delivery to the left hemisphere**. (**A**) Sagittal, (**B**) coronal and (**D**) axial MR views show enhanced T_1_ contrast in the left hippocampus compared to the (**C**) contralateral side. The localized distribution of the signal (arrows) confirms the targeted delivery of Gd(rhodamine-pip-DO3A). The periphery of the brain slices has a high intensity due to the increased contrast applied to the images to visualise the probe.

Three of the mouse brains where Gd(rhodamine-pip-DO3A) was delivered were then used to perform *ex vivo* MRI scans to check whether the probe could be detected by MRI as well as fluorescence microscopy. An increased brightness in T_1_ images was observed within the left hippocampus where the probe had been delivered (Figure 8 A, B, D). The amount of probe detected in the left hippocampus compared to the control right hippocampus was quantified with the normalized signal intensity, which showed a higher intensity detected in the left hippocampus compared to the control regions in all quantified images (Figure S6). After the MRI scans, the brains were sectioned to visualise the location of the fluorescence compared to that of the increased MRI contrast. Higher signal was detected in similar regions in both MRI (Figure 8) and fluorescence (Figure S7) images.

A binding assay of Gd(rhodamine-pip-DO3A) against serum albumin, an abundant protein in the blood, was also performed to assess the interaction of the complex with endogenous albumin in the blood, a factor which can increase the relaxivity of Gd-based contrast agents. Gd(rhodamine-pip-DO3A) was found to interact with albumin as the intrinsic fluorescence emission of serum albumin was quenched upon increasing titration of the probe (Figure S8).

## Discussion

Our biologically compatible imaging agent was designed and prepared by combining a fluorescent moiety with a gadolinium chelate, resulting in a dual-modal compound. The ability to image with both fluorescence microscopy and MRI can provide complimentary information about neuronal morphology and physiology. As a result this ability could be used synergistically to achieve high anatomical resolution and depth penetration (MRI) as well as high sensitivities using optical imaging in preclinical research 60,61.

Our chosen fluorophore, rhodamine B, has been locked into a ring-opened, fluorescent form, which is also cationic to facilitate cellular uptake (Figure 3). For chelate design, one of the main considerations is to ensure that Gd^3+^ ions are not released into a physiological system as these have been shown to be toxic 62. DOTA-derived, macrocyclic ligand systems have been used extensively to chelate metals in biomedical applications, as they are known to form thermodynamically stable and kinetically inert coordination complexes 63. Derivatives of this motif are also the ligand of choice in clinically approved contrast agents such as ProHance, Dotarem and Gadovist and are deemed to be comparatively stable in a physiological context 64-66.

Once prepared and purified, we assessed the photophysical properties of Gd(rhodamine-pip-DO3A) and found that the absorption and emission spectra were dominated by typical rhodamine-based signals (Figure 4) that are compatible with microscopy applications and did not substantially change with pH.

The relaxivity value of the compound was determined as 7.8 mM^-1^ s^-1^ at 400 MHz (25°C), which is higher than the approximately 3-5 mM^-1^s^-1^ usually expected for octadentate gadolinium chelates 67.

Relaxivity values are dependent on parameters such as hydration number, rotational correlation time and water exchange rates, which are influenced by chemical design principles 68. One of the common factors that lead to increased relaxivity values is an increase in molecular weight or rigidity and a corresponding increase in rotational correlation time.

Another possibility is that relaxivity increases are due to the exchange rates of solvent water molecules, which are largely influenced by the local geometry of the macrocycle encapsulating the Gd^3+^ ion. By estimating the SAP to TSAP ratio in the Eu^3+^ analogue 59, we were able to conclude that our compound is likely to have a relatively high TSAP prevalence compared to the parent DOTA complex (~36 % compared to ~15 % for the DOTA complex 69), which may be the cause for the high relaxivity value.

Next, to deliver the compound into the intended brain region, a method using focused ultrasound and microbubbles was employed. This technique has been previously used to transport Gd-based contrast agents into the brain to visualise the increase in BBB permeability following ultrasound treatment 31-33. More recently, ultrasound has been utilized to aid the delivery of liposomes labeled with both gadolinium and rhodamine through the BBB to verify liposome delivery via MRI and optically 70. However, to the best of our knowledge, no optical-MRI probe designed to image neurons has been delivered using this methodology.

In general, Gd-based contrast agents are considered to be extracellular probes due to their hydrophilic nature, however, based on previous studies, we hypothesized that adding a cationic rhodamine derivative to the Gd-DO3A complex would enhance its neuronal uptake 5,16. Specifically, lipophilic and positively charged species typically permeate cellular membranes in response to the membrane potential, which we sought to achieve by incorporating a positive charge 71,72.

As anticipated, Gd(rhodamine-pip-DO3A) was taken up by cells across all ultrasound-treated regions. Regions with less uptake were observed at the edges of the treated regions, showing that the lower ultrasound pressure led to less probe being delivered 44. Cells that showed uptake on the fluorescence channel also showed a darker contrast in the bright field images.

Cellular uptake of Gd(rhodamine-pip-DO3A) was only observed in neurons, while microglia and astrocytes did not show any co-localization with the probe. Neuronal uptake mostly depends on the properties of the probe itself and how cell permeable it is, while microglia and astrocytes will both participate in the removal of unwanted molecules to maintain optimal neural function 73. Microglia are the resident immune cells of the brain and one of their main roles is to phagocytose unwanted molecules, dying or dead cells, and axons to maintain homeostasis in the brain 17. Astrocytes also participate in this process by driving the phagocytic activity of microglia 74,75. From our results, we see that microglia and astrocytes are not uptaking the probe, indicating that neurons are being labeled without these glial cells phagocytosing the probe to remove it from the brain. We suggest that rhodamine could be labeling neurons, not only due to its cationic and lipophilic properties, but also due to a facilitated receptor-mediated uptake. However, in our study we only explored uptake immediately after the ultrasound treatment. In the future, uptake will be explored at later time points as this could be a time-sensitive process. In addition, some of the microglia within the treated regions appear to have a more rounded shape and shorter processes, which is an indication of the microglia being activated 76. To identify astrocytes, we stained brain slices for GFAP, which is a standard method to visualise reactive astrocytes 77. Astrocyte staining was observed very clearly in all treated regions of the brain but not in the opposite control sides, indicating that the astrocytes could be in a reactive state. Such a response could be occurring due to the presence of the probe itself but also in response to the microbubbles stimulating the blood vessels when increasing the permeability of the BBB. As expected, the smaller 1 kDa Gd(rhodamine-pip-DO3A) probe was delivered across a larger region compared to the 3 kDa dextran molecule. Such observation agrees with the size-threshold effect observed using this ultrasound technology 34,38,78. At the edges of the ultrasound-treated region, the ultrasound pressure is lower which would allow the Gd-complex through but not the larger dextran. In addition, the distribution of the probes was found to be significantly different. Gd(rhodamine-pip-DO3A), as would be expected from a smaller probe, was distributed uniformly across the treated region. This is advantageous if interested in labeling all the cells within a specific region or for treatment purposes if a theranostic probe were to be used instead. The dextran, however, was delivered in spot-like patterns, mainly around blood vessels. This difference in distribution could be due to the larger size of the probe but also to the different properties of the compounds themselves, such as surface properties and lipophilicity. When delivering larger compounds such as dextran, a more uniform distribution could be obtained by emitting a different ultrasound sequence, such as rapid short-pulses (RaSP) 44. These shorter pulses have been shown to improve the delivered distribution as well as safety aspects, such as faster blood-brain barrier closing (< 10 min) and reduced albumin extravasation into the brain (3-4 fold less). These short pulses could be the answer to some of the safety issues of this ultrasound technology that have recently been a topic of debate 79-82. Dextran is also eliciting an immune response, with microglial uptake, whilst Gd(rhodamine-pip-DO3A) is not. The larger dextran molecules could be recognized faster by the immune system as being foreign due to their size and other properties.

Detection of the delivered probe by both MRI and fluorescence allows the advantages of both imaging modalities to be exploited when studying neurons *in vivo*. Previous studies have reported a lack of MRI enhancement when using Gd-rhodamine-derived probes 5. In our study, MRI enhancement was detectable in the *ex vivo* brain and both fluorescence and MRI signals were detected in similar regions. However, as the sensitivity of MRI contrast agents is inherently lower than that of optical agents 83, it is possible that not all the regions where the probe was delivered are visible in the MRI images.

Lastly, the probe was found to interact with albumin *in vitro*, which we would expect to occur *in vivo* given that albumin is the most abundant protein in blood 84. The interaction of the probe with albumin would increase its MRI signal 85. We have previously shown that albumin crosses the BBB in certain regions with the same ultrasound parameters as those used in this study 44; however, because albumin is 67 kDa in size, we would expect there to be regions where the probe alone would cross the BBB, whilst albumin would not. Thus, it is possible that we are detecting both the probe itself and the albumin-conjugated probe.

Despite our ability to detect neurons using a dual-modal probe delivered with focused ultrasound, further improvements can be made. In this study, cellular uptake and response to the probe were investigated immediately after the ultrasound-treatment was terminated, allowing the probe under ten minutes to enter the brain and interact with the environment. Looking at these interactions at later time points would enable a more comprehensive understanding of how long the neurons are labeled for and whether the probe would interact with microglia and astrocytes later in time. It could also give insight into how quickly the probe is cleared from the brain. However, the main purpose of this specific dual-modal probe is to facilitate the study of neuronal morphology and physiology rather than for clinical applications, where concerns regarding the presence of free gadolinium in the brain are higher 86. In future work, we will deliver the probe using a RaSP ultrasound sequence, given the evidence of improved efficacy and safety 44. This sequence could improve the immune response, reducing microglial and astrocyte activation.

An alternative method to analyse the fluorescence signal in the different cell types, could be to use flow cytometry. This technique has been shown to separate neurons from glia cells allowing quantitative analysis without the need for transgenic models 87. In the future, we will also investigate the subcellular localization of our synthesized probe, which was detected in the nucleus and cytoplasm with some small high intensity regions. Acquiring higher magnification confocal images of these cells could help identify these high intensity regions. Furthermore, GFAP staining was performed in this study to identify astrocytes. However, GFAP, as mentioned previously, does not label all non-reactive astrocytes as not all these cells express detectable levels of GFAP. In the future we will stain for alternative astrocyte markers to identify non-reactive as well as reactive astrocytes.

Lastly, the location of the probe was detected via MRI in *ex vivo* brains which allows for longer scanning time and therefore higher resolution. *In vivo*, the lower resolution could make the probe more difficult to detect, however, the tissue would not be processed and therefore the scanning would be done sooner compared to this study, where the brains were extracted straight after the treatment and then processed for several days before scanning. Under *in vivo* conditions, waiting longer after the ultrasound treatment could give the probe more time to diffuse and improve the MRI signal. In future work, we intend to detect the probe via *in vivo* MRI and explore some of the applications of this probe, such as studying changes in neuronal density during development and in specific disease states.

## Conclusions

We have herein introduced a method to image neurons with a dual-modal MRI/optical imaging agent delivered non-invasively and locally to the brain using focused ultrasound and microbubbles. We prepared a gadolinium complex combined with a fluorescent rhodamine unit (Gd(rhodamine-pip-DO3A)) and found that its fluorescence and relaxivity properties were suitable for *in vivo* applications. The probe was delivered locally to the left hemisphere of mice using focused ultrasound and microbubbles and was found to spread uniformly (COV = 0.4 ± 0.05). Its cellular uptake was confirmed in neurons, while microglia and astrocytes, although present, did not uptake the probe. Compared to the more conventionally used Texas-Red dextran, the probe, which was substantially smaller in size, had a more uniform distribution and elicited less of an immune response with no microglial uptake. Gd(rhodamine-pip-DO3A) was detected via both fluorescence and MRI *ex vivo*. The delivery of such dual-modal agents into neurons could facilitate the study of neuronal morphology and physiology using the advantages of both imaging modalities.

## Supplementary Material

Supplementary information and figures.

## Abbreviations

^13^C-NMR: carbon nuclear magnetic resonance
^1^H-NMR: proton nuclear magnetic resonance
3D: three dimensions
BBB: blood-brain barrier
BM: Bohr magneton
BODIPY: boron dipyrromethene
br: broad
BSA: bovine serum albumin
COV: coefficient of variation
CPPs: cell penetrating peptides
Cy7: cyanine 7
d: doublet
dB: decibel
DCM: dichloromethane
DO3A: Tetraazacyclododecane-1,4,7-triacetic acid
DOTA: Tetraazacyclododecane-1,4,7,10-tetraacetic acid
ES+: electrospray ionization
FITC: fluorescein isothiocyanate
FLASH: fast low angle shot
Gd: gadolinium
GFAP: glial fibrillary acidic protein
H&E: haematoxylin & eosin
H&L: heavy and light
HeLa: human cervical cancer cells
Hz: hertz
Iba1: ionized calcium binding adaptor molecule 1
IgG: immunoglobulin
IHC: immunohistochemistry
K_SV_: quenching constants
m: multiplet
MALDI: matrix assisted laser desorption/ionization
MIP: maximum intensity projections
MR: magnetic resonance
MRI: magnetic resonance imaging
NeuN: neuronal nuclei
NMR: nuclear magnetic resonance
NOD: normalized optical density
OCT: optimal cutting temperature
PBS: phosphate buffered saline
PC: personal computer
PCD: passive cavitation detector
Pip: piperazine
P_neg_: peak negative pressure
ppm: part per million
q: quartet
r_1_: relaxivity
RT: room temperature
s: singlet
SAP: square antiprism
sbr: broad singlet
t: triplet
T_1_: longitudinal relaxation time
T_E_: echo time
TFA: trifluoroacetic acid
ToF: time of flight
T_R_: repetition time
TSAP: twisted square antiprism
