# Supplementary material for: Neuron labeling with rhodamine-conjugated Gd-based MRI contrast agents delivered to the brain via focused ultrasound
Source: Theranostics. 2020 Feb 3;10(6):2659–74. doi: 10.7150/thno.42665 (PMC7052893; doi:10.7150/thno.42665)
Supplement: Supplementary file 1 — Supplementary information and figures. [file thnov10p2659s1.pdf]

Supplementary Material - Neuron labeling with rhodamine-conjugated Gd-based MRI contrast agents delivered to the brain with focused ultrasound

Synthesis

Tert-butyl-DO3A [81] and compound **1** [48] were prepared via literature methods from commercially available starting materials.

N-(9-(2-(4-(2-chloroacetyl)piperazine-1-carbonyl)phenyl)-6-(diethylamino)-3H-xanthen-3-ylidene)-N-ethylethanaminium (**2**)

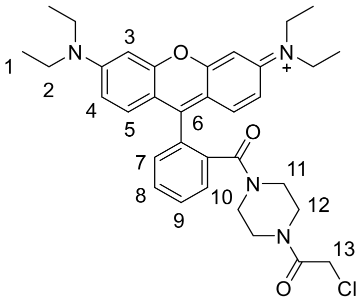

Compound **1** (1.53 g, 2.99 mmol) was dissolved in dichloromethane (50 mL) and cooled to 0°C. NEt_3 (1.67 mL, 11.96 mmol) was added, followed by chloroacetyl chloride (261.8 µL, 3.29 mmol) and the solution was stirred for 3 hours. Water (50 mL) was added to the reaction mixture and the organic layer was separated before extraction of water with dichloromethane (2 x 30 mL). The organic layers were combined, dried over $MgSO_4$, filtered and the solvent was removed under reduced pressure. The crude pink solid was purified by flash column chromatography (DCM/MeOH 100:0 v/v to 95:5 v/v), producing **2** (1.00 g, 62 % yield) as a pink powder.

^{1}H NMR (400 MHz, $CDCl_3$) δ(ppm): 1.26 (12 H, t, $^3J_{H-H}$ = 7.0, C^1H_2), 3.32-3.46 (8 H, m, $C^{11}H_2$ and $C^{12}H_2$), 3.50-3.65 (8 H, m, C^2H_2), 4.13 (2 H, s, $C^{13}H_2$), 6.67-6.72 (2 H, m, C^3H), 6.95-7.02 (2H, m, C^4H), 7.17 (2 H, d, $^3J_{H-H}$ = 9.6, C^5H), 7.26-7.31 (1 H, m, C^7H), 7.46-7.52 (1 H, m, $C^{10}H$), 7.60-7.66 (2 H, m, C^8H and C^9H).

^{13}C NMR (100 MHz, $CDCl_3$) δ(ppm): 12.5 (C^1H_3), 41.2 ($C^{13}H_2$), 41.6 ($C^{11}H_2$ or $C^{12}H_2$), 46.0 (C^2H_2), 47.6 ($C^{11}H_2$ or $C^{12}H_2$), 96.1 (C^3H), 113.7, 114.4 (C^4H), 127.5 ($C^{10}H$), 130.1 (br, C^8H, C^9H and C^7H), 130.2 (br, C^8H, C^9H and C^7H), 131.0, 132.0 (C^5H), 135.0 (C^7-$\underline{C}$-C^6), 155.7, 155.8, 157.7, 165.8 (C13H2-*C*=O), 167.7 (C=O).

MS(ES+) calc. for C34H40ClN4O3 587.2789 $[M+H]^+$, found 587.2792.

N-(6-(diethylamino)-9-(2-(4-(2-(4,7,10-tris(2-(tert-butoxy)-2-oxoethyl)-1,4,7,10-tetraazacyclododecan-1-yl)acetyl)piperazine-1-carbonyl)phenyl)-3H-xanthen-3-ylidene)-N-ethylethanaminium (**3**)

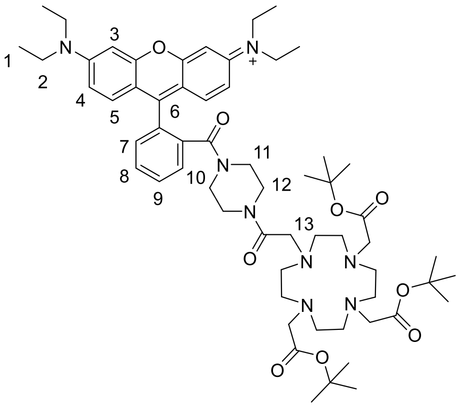

Tert-butyl-DO3A (0.14 g, 0.23 mmol) and compound **2** (0.20 g, 0.34 mmol) were dissolved in acetonitrile (40 mL) and K_2CO_3 (0.127 g, 0.92 mmol) was added. The solution was heated to reflux for 48 hours, cooled and filtered. The solvent was removed under reduced pressure and the crude pink solid was purified by flash column chromatography (DCM/ MeOH 100:0 v/v to 95:5 v/v) to yield **3** (0.16 g, 66 % yield) as a pink solid.

^{1}H NMR (400 MHz, $CDCl_3$) δ(ppm): 1.28 (12 H, t, $^3J_{H-H}$ = 7.0, C^1H_3), 1.40 (18 H, s, tBu), 1.42 (9 H, s, tBu), 1.70-2.68 (24 H, m), 3.23-3.40 (8 H, m, $C^{11}H_2$ and $C^{12}H_2$), 3.51-3.67 (8 H, m, C^2H_2), 6.73-6.77 (2 H, m, C^3H), 6.96-7.03 (2 H, m, C^4H), 7.15 (2 H, d, $^3J_{H-H}$ = 9.2, C^5H), 7.22-7.25 (1 H, m, C^7H), 7.42-7.46 (1 H, m, C^{10}H), 7.60-7.65 (2 H, m, C^8H and C^9H).

^{13}C NMR (100 MHz, $CDCl_3$) δ(ppm): 12.6 (C^1H_3), 27.9 (CH_3 tBu), 41.1, 41.7, 44.4 (br, $C^{11}H_2$ and $C^{12}H_2$), 46.1 (C^2H_2), 47.6, 48.4 (br), 52.7 (br), 54.9, 55.7, 81.4 (tBu), 81.8 (tBu), 96.2 (C^3H), 113.8, 114.6 (C^4H), 127.4 (C^{10}H), 130.1 (C^7H, C^8H or C^9H), 130.2 (C^7H, C^8H or C^9H), 130.9 (C^7H, C^8H or C^9H), 131.3, 132.0 (C^5H), 135.1 (C^7H-$\underline{C}$-C^6), 155.4, 155.8, 157.8, 168.1 (Rh-C=O), 170.6 (C=O), 172.6 (C=O) 172.7 (C=O).

MS(ES+) calc. for C48H65N8O9 897.4875 $[M+H]^+$, found 897.4906.

*N-(6-(diethylamino)-9-(2-(4-(2-(4,7,10-tris(carboxymethyl)-1,4,7,10-tetraazacyclododecan-1-yl)acetyl)piperazine-1-carbonyl)phenyl)-3H-xanthen-3-ylidene)-N-ethylethanaminium (**4**)*

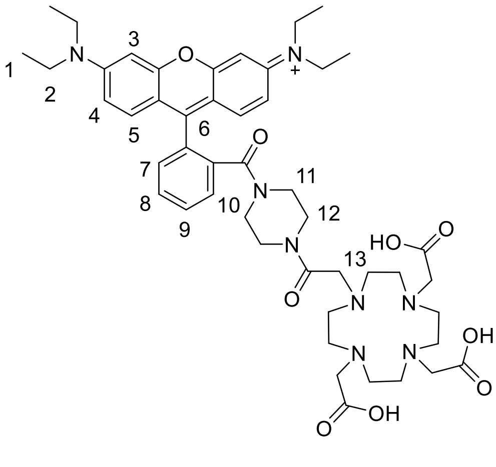

Compound **3** (101.6 mg, 95.3 μmol) was treated with trifluoroacetic acid/dichloromethane (1/1 *v/v*, 4 mL) and stirred for 16 hours at room temperature. The solvent was removed under reduced pressure and the residue purified by reverse phase flash column chromatography (H_2O/MeOH/ TFA 95:5:0 v/v to 0:99.9:0.1 v/v), yielding **4** (63.5 mg, 74 % yield) as a pink solid.

^{1}H NMR (400 MHz, CD_3OD) δ(ppm): 1.30 (12 H, t, $^3J_{H-H}$ = 7.2, C^1H_3), 3.04-3.14 (8 H, m, CH_2), 3.36-3.55 (10 H, m, CH_2), 3.64-3.73 (12 H, m, C^2H_2 and CH_2), 6.95 (2 H, d, $^3J_{H-H}$ = 2.4, C^3H), 7.05-7.11 (2

H, m, C^4H), 7.29 (2 H, d, $^3J_{H\text{-}H}$ = 9.6, C^5H), 7.48-7.49 (1 H, m, C^7H), 7.72-7.79 (3 H, m, C^8H, C^9H and $C^{10}H$).

^{13}C NMR (100 MHz, CD_3OD) δ(ppm): 12.9 (C^1H_3), 42.8 (br), 44.9, 45.4, 45.7, 46.9 (C^2H_2), 50.7 (br), 52.2 (br), 52.7 (br), 54.4 (br), 56.5, 57.1, 97.3 (C^3H), 114.8, 115.4 (C^4H), 129.0 ($C^{10}H$), 131.2 (C^8H or C^9H), 131.3(C^8H or C^9H), 131.7 (C^7H), 132.1, 133.2 (C^5H), 136.6 (C^7H-*C*-C^6), 157.0, 157.2, 159.2, 169.5 (C=O), 170.0 (C=), 170.8 (C=O), 176.5 (C=O).

MS(ES+) calc. for C48H65N8O9 897.4875 $[M+H]^+$, found 897.4906.

Compound **5**

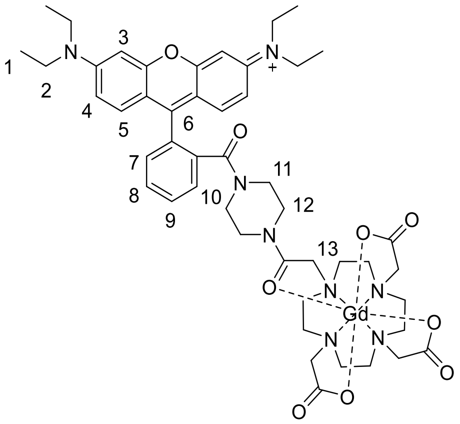

Compound **4** (33.8 mg, 38.0 μmol) and $GdCl_3.6H_2O$ (28.2 mg, 75.9 μmol) were dissolved in H_2O (10 mL) and the pH was adjusted to 5.5 using 1 M NaOH. The solution was stirred for 16 hours at room temperature, following which the solvent was removed under reduced pressure and the residue was purified by reverse phase flash column chromatography (H_2O/ MeOH/ TFA 95:5:0 v/v to 0:99.9:0.1 v/v), yielding **5** (29.4 mg, 74 % yield) as a pink solid.

MS(ES+) calc. for C48H62N8O9Gd 1052.3881 $[M+H]^+$, found 1052.3900.

H&E Staining

Brains were paraffin-embedded and 1.2 mm were then trimmed from the top of the brain to reach the hippocampus. Eight sections were acquired for each level (12 levels in total), spanning across 48 µm of brain tissue at each level. In between each level, 80 µm were discarded. The first two sections of each level were stained for H&E and were analysed for any signs of red blood cell extravasation, microvacuolations and dark neurons.

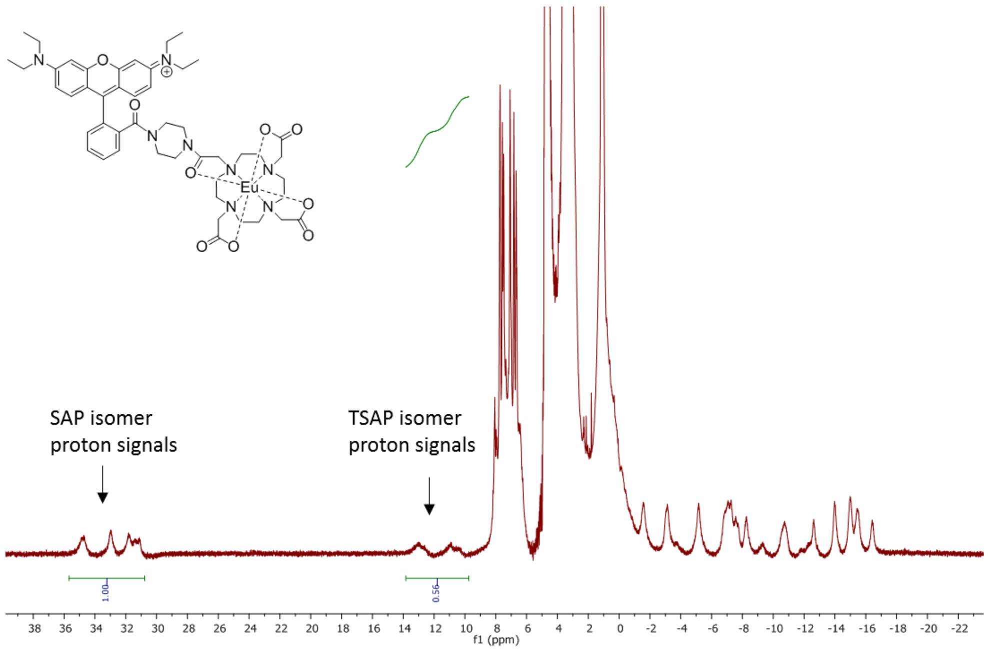

Figure S1. ^{1}H-NMR spectrum of Eu(rhodamine-pip-DO3A), run in D_2O at 500 MHz and 298 K, displaying typical resonances for the square antiprism (SAP) isomer (shift range 30 - 35 ppm) and the less intense twisted square antiprism (TSAP) isomer (shift range 10 - 14 ppm).

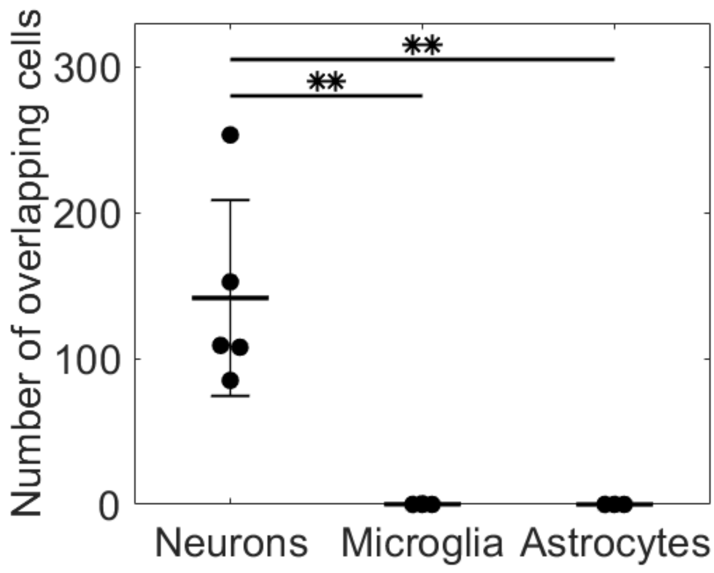

Figure S2. Number of neurons, microglia and astrocytes overlapping with Gd(rhodamine-pip-DO3A). Quantification performed on stained brain slices (10x) shows that the number of neurons that overlap with the probe is significantly different from the number of microglia and astrocytes overlapping with the probe (*P < 0.01*).

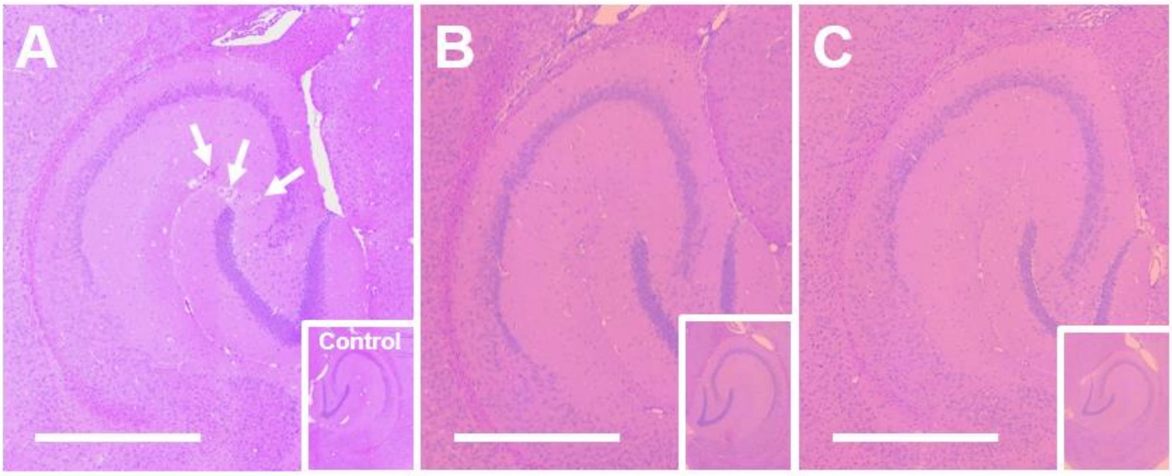

Figure S3. H&E staining to assess damage. Representative images are shown from each of the three brains stained with H&E (10x). The left targeted hippocampus is shown with the right hippocampus in the white box on the bottom right of each image. (A) This was the only brain out of the three where small regions of red blood cell extravasation and microvacuolations were observed (white arrows). (B-C) In these two brains no signs of damage were observed. The white scale bar in the images indicates 1000 µm.

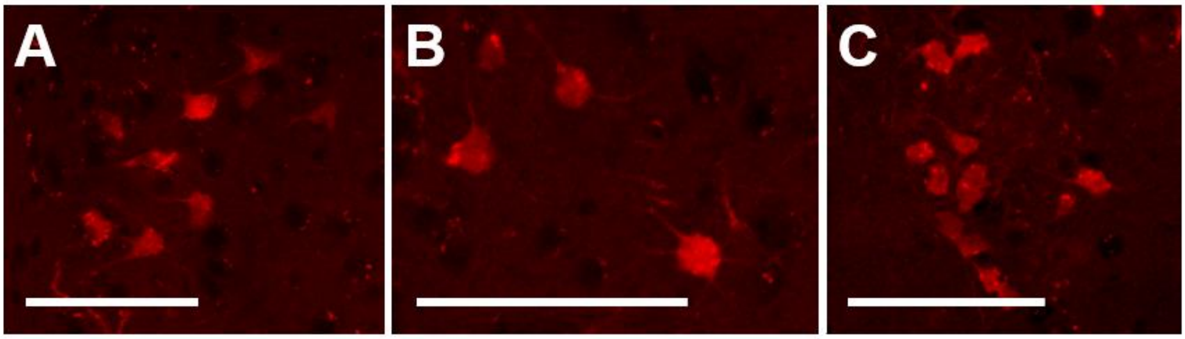

Figure S4. Subcellular localization of Gd(rhodamine-pip-DO3A) within neuronal-like cells. Confocal microscopy images (20x) of brain regions with Gd(rhodamine-pip-DO3A) delivery. The probe appears to be localized within the nucleus and cytoplasm, with small high intensity regions visible within the cell. The white scale bars in these images indicate 100 µm.

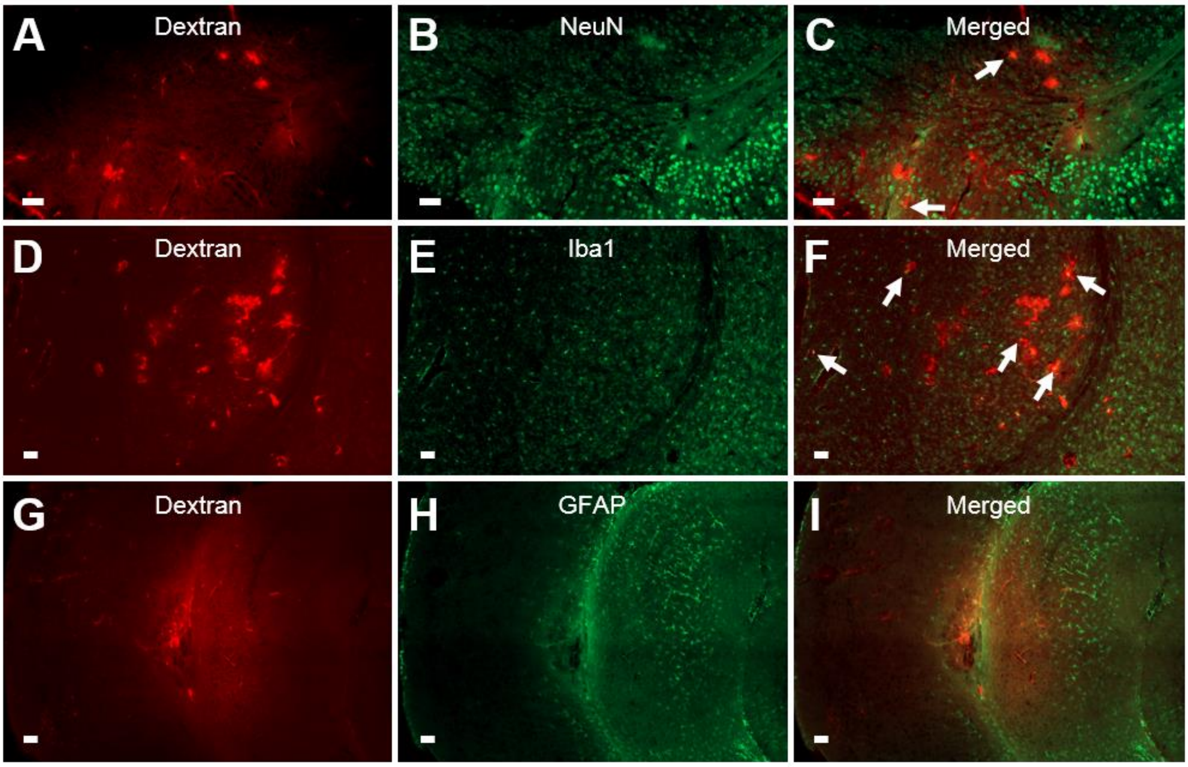

Figure S5. Neuronal, microglial and astrocyte staining of brain slices with dextran delivery. Fluorescence images (10x) of dextran delivery (A, D, G) and of immunohistological staining for neurons with NeuN (B), microglia with Iba1 (E) and astrocytes with GFAP (H), with the respective merged channels (C, F, I). White arrows indicate overlap between dextran and cells. Overlap was observed with neurons and more so with microglia. No overlap was observed with astrocytes. The white scale bars indicate 50 µm.

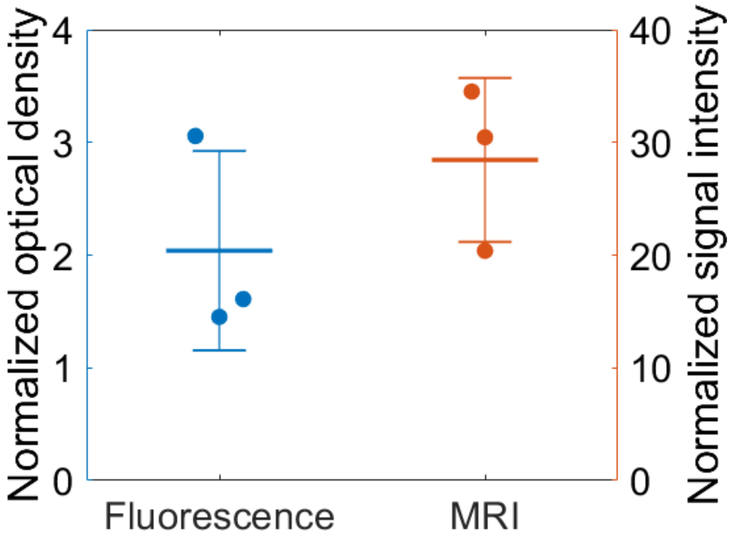

Figure S6. Increased fluorescence and MRI signal in the left hemisphere compared to the control region. Normalized optical density (NOD) measurements from fluorescence images (performed on 6 slices per brain) and normalized signal intensity measurements from MRI images (performed on 5 slices per brain) show that the signal detected is always higher in the left targeted hemisphere compared to the right control hemisphere (zero line). Measurements were taken on the same three brains.

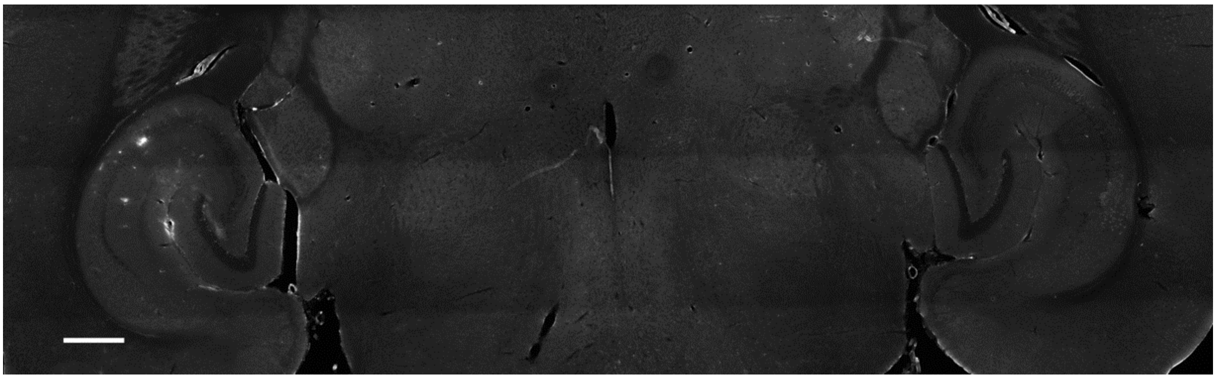

Figure S7. Fluorescence detection of Gd(rhodamine-pip-DO3A) in brain scanned with MRI. Fluorescence image (10x) shows that Gd(rhodamine-pip-DO3A) was detected optically in similar regions to those detected by MRI in the left hippocampus (see Figure 8). In the right hippocampus (control region) no compound was detected. The white scale bar indicates 500 µm.

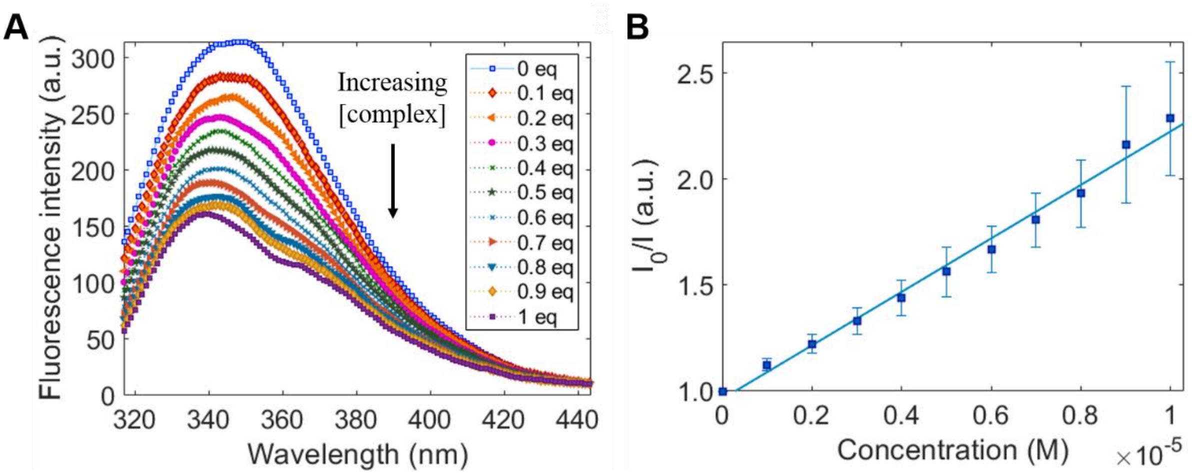

Figure S8. Binding of Gd(rhodamine-pip-DO3A) to albumin. (A) Emission spectra show how the fluorescence intensity of albumin decreases with increasing amount of Gd(rhodamine-pip-DO3A), indicating that an interaction between albumin and the complex is taking place (a.u. = arbitrary units; eq = equivalents). **(B)** To obtain the rate of quenching a Stern-Volmer plot was obtained showing the rate of fluorescence without the complex (I_0) over the rate of fluorescence with the complex (I) as the concentration of the complex varies (quenching constant K_{sv} = 1.26 x 10^5).
